# 254 What You Don't Know Can Hurt You: Investigation of Acute Hepatitis B in an Outpatient Clinic- Connecticut, 2025

**DOI:** 10.1017/ash.2026.10622

**Published:** 2026-06-23

**Authors:** Emily Chasco, Kimberly Dukes, Sarah Adams, Loreen Herwaldt

**Affiliations:** 1 Institute for Clinical and Translational Science, University of Iowa; 2 Dept of Gen Int Med, Carver College of Medicine, University of Iowa; 3 Department of Internal Medicine, Carver College of Medicine, University of Iowa, IA, United States; 4 University of Iowa Carver College of Medicine

## Abstract

**Background:** Recent research has investigated the role of sinks (including sink taps, filters, water traps, etc.) in the spread of multidrug-resistant organisms (MDROs) and other pathogens. As part of a larger study on hand hygiene (HH) and personal protective equipment (PPE) adherence, we explored healthcare professionals’ (HCP’s) interactions with water and wet surfaces during patient care processes in intensive care units (ICUs). **Methods:** We conducted ethnographic observations of HCP’s patient care processes in the medical and surgical ICUs of a large academic hospital, and we conducted mini-interviews (~ 3-7 minutes) with a subset of these HCP. Observations focused on HH and PPE adherence during contaminating tasks. We also captured additional relevant data (e.g., room layouts, care activities, fomite/device interactions). We documented observations and interview responses in fieldnotes that we imported into MAXQDA qualitative software and coded activities using a combined deductive-inductive approach. We conducted data collection and analyses iteratively, with early analysis informing later data collection. HCP interactions with water emerged inductively as a topic of interest during this process. **Results:** Between 3/2022-5/2023, we observed 104 HCP engaged in patient care and conducted 31 mini-interviews. Interactions with sinks and sink-adjacent counters were common. We observed HCP placing pillows and other items on wet counters, water remaining in sinks after use, HCP contacting water droplets on sink walls and counters, and HCP setting items (e.g., IV bags, used towels) in the sink that they later carried to trash or laundry bins. HCP also leaned against surfaces, including sink-adjacent counters and patient-adjacent areas (e.g., beds, bed rails). Such activities could transfer contaminated water droplets to HCP’s clothing, floors, or other surfaces. We also observed water splashing due to sink design or the water pressure when HCP did activities like filling drinking cups or rinsing containers. During mini-interviews, HCP discussed their preference for soap-and-water handwashing (versus hand sanitizer) in specific situations, which they often performed at the same in-room sinks they used during patient care activities as outlined above. Splash back from sink drains could spread pathogens to HCP’s hands. **Conclusion:** Our study focused on HH and PPE adherence, therefore, we did not systematically collect observations on water in ICUs. Nonetheless, we observed HCP interacting with sinks, water, and wet surfaces in ways that highlight the potential for water-borne pathogen transmission. Infection prevention programs and hospitals should consider these interactions when developing infection prevention guidelines and designing construction and renovation projects.

